# Iron Oxide (Magnetite)-Based Nanobiomaterial with Medical Applications—Environmental Hazard Assessment Using Terrestrial Model Species

**DOI:** 10.3390/jox14010017

**Published:** 2024-02-22

**Authors:** Susana I. L. Gomes, Janeck J. Scott-Fordsmand, Mónica J. B. Amorim

**Affiliations:** 1 Department of Biology & CESAM, University of Aveiro, 3810-193 Aveiro, Portugal; susana.gomes@ua.pt; 2 Department of Ecoscience, Aarhus University, C.F. Møllers Alle 4, DK-8000 Aarhus, Denmark; jsf@bios.au.dk

**Keywords:** advanced materials, standard tests, long term, LUFA 2.2 soil, *Enchytraeus crypticus*, *Folsomia candida*

## Abstract

Nanobiomaterials (NBMs) have tremendous potential applications including in cancer diagnosis and treatment. However, the health and environmental effects of NBMs must be thoroughly assessed to ensure safety. Fe_3_O_4_ (magnetite) nanoparticles coated with polyethylene glycol (PEG) and poly (lactic-co-glycolic acid) (PLGA) were one of the focus NBMs within the EU project BIORIMA. Fe_3_O_4_ PEG-PLGA has been proposed to be used as a contrast agent in magnetic resonance imaging for the identification of solid tumors and has revealed low cytotoxicity in several cell lines. However, the effects of Fe_3_O_4_ PEG-PLGA have not been assessed in terrestrial environments, the eventual final sink of most materials. In the present study, the effects of Fe_3_O_4_ PEG-PLGA and its precursor, (un-coated) Fe_3_O_4_ NMs, were assessed in soil model invertebrates *Enchytraeus crypticus* (Oligochaeta) and *Folsomia candida* (Collembola). The endpoints were survival, reproduction, and size, based on the standard OECD test (28 days) and its extension (56 days). The results showed no toxicity for any of the endpoints evaluated, indicating that the NBM Fe_3_O_4_ PEG-PLGA poses no unacceptable risk to the terrestrial environment.

## 1. Introduction

The application of nanotechnology into the field of biotechnology to create nanobiomaterials (NBMs)—nanoscale materials with a high biocompatibility—has the potential to revolutionize the biomedical industry due to a vast list of applications. Among the possible applications are tissue repair and regeneration, drug and gene delivery, cancer therapy, medical imaging, etc. [1,2,3,4,5,6,7,8,9,10]. However, despite their huge potential, concerns regarding the possible health and environmental risks of NBMs must be assessed [11,12,13]. This is a transnational issue, addressed by the EU H2020-funded BIORIMA project—BIOmaterial RIsk MAnagement (GA No. 760928). This project aimed to develop an integrated risk management framework for the NBMs used in advanced therapy medicinal products and medical devices. One of the NBMs produced and tested within BIORIMA consisted of Fe_3_O_4_ (magnetite) nanoparticles coated with polyethylene glycol (PEG) and poly (lactic-co-glycolic acid) (PLGA), referred to as Fe_3_O_4_ PEG-PLGA. This NBM has been proposed to be used as a contrast agent in magnetic resonance imaging for the identification of solid tumors [14,15].

Studies on NBMs’ toxicity, including Fe_3_O_4_ PEG-PLGA, are mostly available for in vitro models, without much information on their effects on environmental species. Several in vitro cytotoxicity studies with Fe_3_O_4_ PEG-PLGA indicate an overall low-to-no toxicity to different cell models [16,17,18]. Genotoxicity was also not observed in HCT116 cells exposed to Fe_3_O_4_ PEG-PLGA up to 50 μg/mL [16,18], despite the increase in reactive oxygen species (ROS) levels observed above 10 μg/mL [16]. In a study performed in an environmental species, i.e., using the fish cell lines RTL-W1 (CRL-2301, derived from the liver), RTgill-W1 (CRL-2523, derived from the branchial arc), and RTS-11 (derived from the spleen), up to 100 μg/mL within 24 h [19] of Fe_3_O_4_ PEG-PLGA was also not acutely toxic. However, when the exposure of RTgill-W1 cells was prolonged to 28 days, cytotoxicity occurred: for instance, a 20% effect on the cell membrane (1.6 days), mitochondrial activity (16 days), and the lysosomes (19 days). Nevertheless, there was an almost total recovery of the cells exposed for 14 days and transferred to a clean medium for up to 28 days, except at the lysosomal level [19]. Finally, the occupational risks of Fe_3_O_4_ PEG-PLGA were determined to be negligible for the workers dealing with this NBM along its life cycle stages (i.e., product manufacturing, use, and end of life) [14]. However, all materials, including NBMs, will eventually reach the environment by being released during production and usage, by accidental spills, or, ultimately, through waste disposal at their end of life [13,20]. Thus, assessing the possible environmental effects (or lack of effects) of NBMs is essential to ensure their safety and introduction to the market.

Hence, the aim of the present study was to investigate the environmental hazards of Fe_3_O_4_ PEG-PLGA and compare it to the uncoated Fe_3_O_4_ NM using two soil invertebrates from distinct groups (Arthropoda and Annelida), i.e., the species *Enchytraeus crypticus* (Enchytraeidae, Annelida) and *Folsomia candida* (Collembola, Arthropoda). The selected test species have similar standardized one-generation tests, with28 days of exposure duration [21,22], and also well-developed longer-term tests, 56 days in duration [23,24]. This enables the comparison between species during the same exposure periods, also covering long(er)-term exposure, as recommended for NMs. Further, enchytraeids and collembolans cover different routes of exposure and life traits in soil. Thus, in our study, the effects were assessed via LUFA 2.2 soil exposure, based on the OECD standard (28 days) reproduction tests and its extension, with a longer-term exposure (56 days).

## 2. Materials and Methods

### 2.1. Test Species

*Enchytraeus crypticus* (Westheide & Graefe, 1992) cultures were kept under controlled temperature (20 ± 2 °C) and photoperiod (16:8 h, light–dark) conditions in an agar medium. This medium consisted of sterilized Bacti-Agar medium (Oxoid, Agar No. 1) and a mixture of four different salt solutions at the final concentrations of 2 mM CaCl_2_·2H_2_O, 1 mM MgSO_4_, 0.08 mM KCl, and 0.75 mM NaHCO_2_. Food (ground autoclaved oats) was provided twice per week. The cultures were synchronized to obtain 18–20-day-old organisms (for further details on culture synchronization see [25]). 

*Folsomia candida* (Willem, 1902) cultures were kept on a moist substrate of plaster of Paris and activated charcoal (8:1 ratio) at 20 ± 2 °C and a photoperiod of 16:8 h (light–dark). Food (dried baker’s yeast (Saccharomyces cerevisiae)) was provided once per week. The cultures were synchronized to obtain 10–12-day-old organisms.

### 2.2. Test Soil

The experiments were performed using natural standard LUFA 2.2 soil (LUFA Speyer, Speyer, Germany). The soil’s main characteristics can be summarized as follows: pH (0.01 M CaCl_2_) = 5.6 ± 0.4; organic carbon = 1.71 ± 0.30%; cation exchange capacity (CEC) = 9.2 ± 1.4 meq/100 g; maximum water-holding capacity (maxWHC) = 44.8 ± 2.9 g/100 g; and texture = 8.0 ± 1.5% clay, 13.7 ± 1.0% silt, and 78.3 ± 1.0% sand content.

### 2.3. Test Materials

An iron (II, III) oxide nanomaterial, Fe_3_O_4_ (Sigma-Aldrich, 97% trace metals basis, Merck KGaA, Darmstadt, Germany)—a nanopowder with 50–100 nm particle size (SEM)—was used as purchased and further referred to as Fe_3_O_4_ NM. Fe_3_O_4_ NM is the precursor of the nanobiomaterial (NBM) Fe_3_O_4_ PEG-PLGA.

The iron (II, III) oxide NBM—Fe_3_O_4_ PEG-PLGA, a suspension—was synthesized as described in [15,26] and provided in the framework of the BIORIMA research project (H2020-NMBP-2017, GA No. 760928). It consists of the precursor Fe_3_O_4_ NM (0.3 wt%) coated with a block copolymer containing two polymeric units of polyethylene glycol (PEG) and biocompatible block copolymer containing two polymeric units of polyethylene glycol (PEG) and poly (lactic-co-glycolic acid) (PLGA), and a diameter of around 15 nm. Briefly, Fe_3_O_4_ NM suspended in diethylene glycol was superficially functionalized with [N-(3,4-dihydroxyphenethyl) dodecanamide (DDA)] and dispersed in THF; after this, a THF solution of PGLA-b-PEG-COOH block copolymer was added to the Fe_3_O_4_ NM–DDA suspension. The formation of the hybrid Fe_3_O_4_ PEG-PLGA was achieved by means of the nanoprecipitation method: two streams of fluid (1. organic dispersion of functionalized magnetite and PLGA-b-PEG-COOH and 2. phosphate-buffered solution in a volumetric ratio of 1/10) were mixed under a constant flux into a mixing cell with vigorous stirring. The so-formed dispersion was then dialyzed (Cogent M system, Pellicon membrane 2 Mini, cut-off 100 kDa) to remove the organic phase using a pure phosphate-buffered aqueous solution. The system was then concentrated to a final concentration of 0.3 wt% (Fe_3_O_4_) and filtered through a syringe filter (Millipore Sterivex, 0.22 μm, polyethersulfone membrane). The dispersant of the suspension (similar formulation without the Fe_3_O_4_ particles) was tested alone as the control dispersant.

### 2.4. Materials Characterization

Fe_3_O_4_ NM and Fe_3_O_4_ PEG-PLGA were characterized in terms of size and surface charge. The Fe^2+/3+^ dissolution from the nanoparticles was determined for Fe_3_O_4_ PEG-PLGA. The samples corresponded to aqueous suspensions of Fe_3_O_4_ NM or Fe_3_O_4_ PEG-PLGA. The hydrodynamic diameters and zeta-potentials were assessed using a Zetasizer instrument (Zetasizer Nano-ZS, Malvern Instruments, Worcestershire, UK). The results referring to the intensity signal were obtained by averaging three measurements. 

Static dissolution measurements were performed to assess the release of Fe^2+/3+^ from the Fe_3_O_4_ PEG-PLGA nanoparticles. The samples were filtered through 10 kDa molecular weight cut-off membranes (centrifuge cycle: 5000 rpm for 40 min), and the filtrated solution was analyzed by ICP-OES coupled with a OneNeb nebulizer (ICP-OES 5100, vertical dual-view apparatus from Agilent Technologies, Santa Clara, CA, USA). The analysis was performed in a radial viewing mode, and the calibration curves were obtained with 0.05, 0.1, 1.0, 10.0, and 100.0 mg/L standards for the Fe element. Nitric acid was added both to the standards and the diluted samples (1:10 *v*/*v*). The concentration of ions was directly evaluated by ICP-OES determination. The results from the ICP-OES were reported as the average of three independent measurements with the relative standard deviation (RSD). 

The redox activity of Fe_3_O_4_ PEG-PLGA was also evaluated: this was achieved, at first, by electronic paramagnetic resonance (EPR) spectroscopy in 0.1 M of phosphate buffer and a pH of 7.4, assessing ROS generation from water or dissolved oxygen, then by Fenton-like reactions (from hydrogen peroxide), free radical species by C-H bond cleavage (probe molecule sodium formiate HCOONa), and, finally, free radical species by O-H bond cleavage (probe molecule TEMPONE-OH). 

The experiments performed were also designed to assess whether the reactivity was due to the particles or to iron ions possibly released into the solution. This was achieved by testing the presence of free radicals in the presence of the NBM or by incubating the NBM in the appropriate fluid and performing the experiment on the supernatant after centrifugation or filtration. The method for the investigation of OH radical generation assumes that the sample does not have any radical scavengers, and it can be assumed that all the OH radicals generated by the nanomaterials react with salicylic acid to give hydroxylation products. The production of OH radicals was evaluated in suspensions containing the nanomaterial under investigation at a concentration of 100 ppm, 10 mM of phosphate buffer (pH 7.4), 50 µM of salicylic acid, and 10 µM of hydrogen peroxide. The suspensions were kept under magnetic stirring for 24 h. The samples were filtered and analyzed by HPLC equipped with a C18 column and a fluorescence detector. OH radicals’ estimation was carried out considering the amount of 2,5 di-hydroxybenzoic acid determined in suspension after 24 h.

### 2.5. Spiking Procedures

The concentrations tested were 0, 100, 200, 500, 1000, and 3200 mg Fe/kg soil dry weight (DW) for Fe_3_O_4_ NM and 0, 10, 100, 200, and 500 mg Fe/kg soil DW for Fe_3_O_4_ PEG-PLGA. For Fe_3_O_4_ PEG-PLGA, the 500 mg Fe/kg soil corresponded to the maximum concentration achievable in soil, given the concentration of the delivered stock suspension (0.3 wt% Fe_3_O_4_). The dispersant of Fe_3_O_4_ PEG-PLGA was tested alone, using the same amount present in the highest tested concentration.

For Fe_3_O_4_ NM, the spiking followed the guideline recommended for solid/powder nanomaterials in soil [27], with each replicate prepared individually to ensure total raw amounts of the tested material. In short, dry powders of the NM were mixed manually with dry soil to obtain the corresponding concentration range. After that, deionized water was added to reach 50% of the soil’s maxWHC. The soil was homogeneously mixed and left to equilibrate for 1 day prior to the start of the test.

For Fe_3_O_4_ PEG-PLGA (and its dispersant), the stock suspension (as synthesized) was serially diluted using MQ water to obtain the desired test concentrations. The spiking followed the guidelines for nanomaterials [27]. In short, the prepared suspensions were added to the pre-moistened soil to reach 50% of the soil’s maxWHC, with each replicate being prepared individually to ensure total raw amounts of the tested material. The soil was homogeneously mixed and left to equilibrate for 1 day prior to the start of the tests.

### 2.6. Test Procedures

#### 2.6.1. *Enchytraeus crypticus*

The tests followed the standard guideline for the Enchytraeid reproduction test [21] (28 days), plus the OECD extension (56 days), as described in [24]. In summary, the standard test was extended for an additional 28 days (56 days in total) and by adding extra monitoring sampling times at days 7, 14, 21, 28, and 56. The endpoints for sampling were survival at days 7, 14, 21, 28, and 56 and reproduction and size at days 28 and 56. Four replicates per treatment were carried out, except for days 7, 14, and 21, which all had one replicate. The test started with ten synchronized-age animals (18–20 days after cocoon laying) per test vessel containing moist soil (⌀4 cm test vessel with 20 g of soil for exposure up to day 28, and ⌀5.5 cm test vessel with 40 g of soil for exposure up to day 56) and a food supply (22 ± 2 mg, autoclaved rolled oats). The test ran at 20 ± 1 °C and a 16:8 h photoperiod. Food (11 ± 1 mg until day 28 and 33 ± 3 mg from day 28 to day 56) and water were replenished weekly. On sampling days 7, 14, 21, and 28, the adults were carefully removed from the soil and counted to assess survival. The juveniles were counted on days 28 and 56 using a stereo microscope to assess reproduction, after being fixed for 24 h with ethanol and Bengal rose (1% in ethanol). For the replicates which continued until day 56, the adults were carefully removed from the soil on day 28. The adult animals collected on day 28 were photographed to assess their size (length), using the software ImageJ (v.1.52a, Wayne Rasband, U. S. National Institutes of Health, Bethesda, MD, USA).

#### 2.6.2. *Folsomia candida*

The tests followed the standard guideline for the Collembolan reproduction test in soil [22] (28 days), plus the OECD extension (56 days), as described in [23], which represents one additional generation in comparison to the standard. In summary, the standard test was extended for an additional 28 days (56 days in total) and by adding extra monitoring sampling times at days 7, 14, 21, 28, and 56. The endpoints assessed were survival, reproduction, and size at all sampling times. Four replicates per treatment were carried out, except for days 7, 14, and 21, which all had one replicate. The test started with ten synchronized-age animals (10–12 days after hatching) per test vessel containing moist soil (⌀5.5 cm test vessel with 30 g of soil) and food supply (2–10 mg, baker’s yeast). The test ran at 20 ± 1 °C and a 16:8 h photoperiod. Food and water were replenished weekly. At each sampling day (days 7, 14, 21, 28, and 56), the test vessels were flooded with water, the content was transferred to a crystallizer dish, and the surface was photographed for further analyses (count and measure (size, area)) using the software ImageJ (v.1.52a, Wayne Rasband, National Institutes of Health, USA). For the replicates which continued until day 56, after a similar flooding and photographing procedure, ten of the biggest juveniles (ca. 11 days old) were transferred to new test vessels containing soil (spiked at day 0) for an additional 28 days of exposure. On day 56, survival (F1), reproduction (F2), and size were assessed, following the previously described procedure.

### 2.7. Data Analysis

The differences between the control and the treatments were assessed for all the endpoints (survival, reproduction, and size) using a one-way analysis of variance (ANOVA) followed by the Dunnett’s post hoc test (*p* < 0.05). For the Fe_3_O_4_ PEG-PLGA data, the control and the dispersant were compared using a *t*-test (*p* < 0.05) (SigmaPlot v.14.0, Systat Software, Inc., San Jose, CA, USA). The effect concentrations (ECx) were estimated modeling data to a logistic 2 parameters’ regression, using the Toxicity Relationship Analysis Program software (TRAP 1.30a, USEPA).

## 3. Results

### 3.1. Materials Characterization

Fe_3_O_4_ NM had a strong agglomeration in water, as noted by its high hydrodynamic diameters and PDI (Table 1 and Table S1), indicative of the high instability of the system. The particles presented a negative Z-potential that decreased with declining concentrations (Table 1 and Table S1), indicating an increased stability at lower concentrations. 

The Fe_3_O_4_ PEG-PLGA suspension had a hydrodynamic diameter of about 75 nm and a negative Z-potential (around −50 mV), consistent with the stability provided by the PEG-PLGA coating. There was no difference across concentrations. The static dissolution performed highlighted little solubility of the nanoparticles, which did not exceed the detection limit of the ICP instrument (0.01 mg Fe/L) (Table 1).

Fe_3_O_4_ PEG-PLGA did not generate ROS from water, dissolved oxygen, or hydrogen peroxide (Fenton-like reaction). It was not able to cleave the C-H bond of sodium formiate but generated oxygen-centered radicals from the probe TEMPONE-OH. The reactivity was due to the particles and not free iron ions.

Fe_3_O_4_ PEG-PLGA was able to produce OH radicals (Table S2), evidenced by a 2.5 di-hydroxybenzoic acid (2.5 DBHA) concentration higher than the detection limit, calculated as a concentration equivalent to the “blank” signal plus three times the standard deviation of the calibration line intercept. Fe_3_O_4_ PEG-PLGA showed reduction and oxidation waves in water.

### 3.2. Ecotoxicity Tests

For the *E. crypticus* tests, the validity criteria were fulfilled, within the standard OECD guideline [21]: that is, adult mortality was below 20%, and the number of juveniles was higher than 50 per replicate in the controls, with a coefficient of variation lower than 50%. Similarly, the validity criteria were fulfilled for the *F. candida* tests [22]: that is, adult mortality was below 20%, and the number of juveniles was higher than 100 per replicate in the controls, with a coefficient of variation lower than 30%. The soil pH had little variation between the test treatments or over time (Tables S3 and S4).

For the Fe_3_O_4_ PEG-PLGA tests, there were no significant differences between the control (moist LUFA 2.2 soil) and the control dispersant; thus, the data were pooled for the graphs and the statistical analysis.

**Figure 1:**
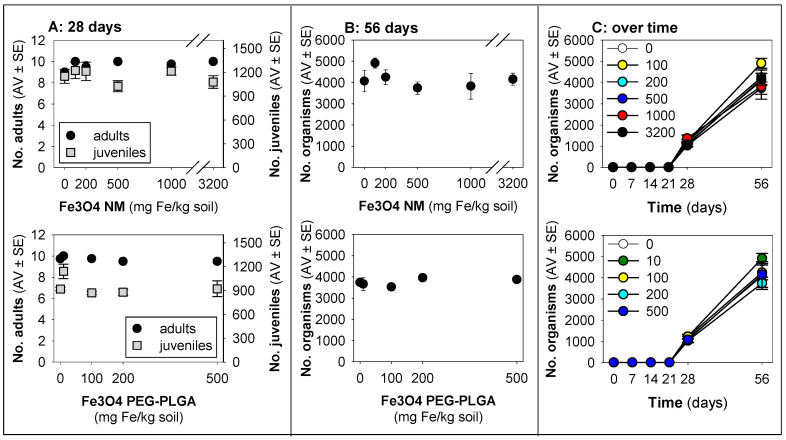
Results in terms of survival and reproduction when exposing *Enchytraeus crypticus* in LUFA 2.2 soil to Fe_3_O_4_ NM and Fe_3_O_4_ PEG-PLGA over (**A**) 28 days (OECD standard) and (**B**) 56 days (OECD standard extension), and (**C**) overview of the time series sampling on days 7, 14, 21, 28, and 56. The values represent the number of adults, juveniles, and population as the average ± standard error (AV ± SE). An enlarged version of panel (**C**) is presented in Figure S2.

For *E. crypticus*, no effect on survival or reproduction caused by either Fe_3_O_4_ NM or Fe_3_O_4_ PEG-PLGA was observed (Figure 1), although a small increase in performance occurred at lower concentrations, a hormesis-like phenomenon. There were no effects on size (Figure S1).

**Figure 2:**
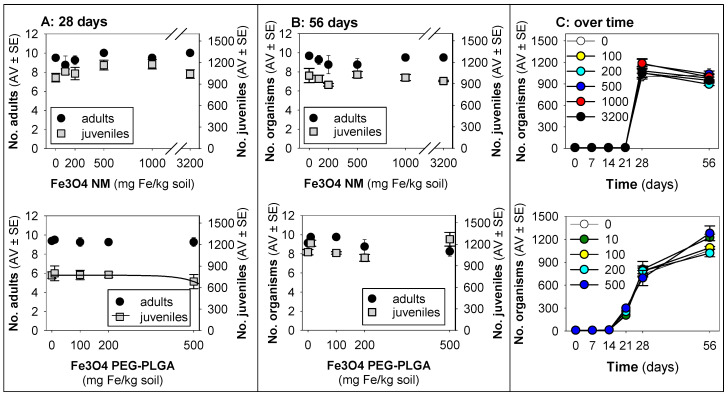
Results in terms of survival and reproduction when exposing *Folsomia candida* in LUFA 2.2 soil to Fe_3_O_4_ NM and Fe_3_O_4_ PEG-PLGA over (**A**) 28 days (OECD standard) and (**B**) 56 days (OECD standard extension), and (**C**) overview of the time series sampling on days 7, 14, 21, 28, and 56. The values are expressed as the average ± standard error (AV ± SE). An enlarged version of panel (**C**) is presented in Figure S3.

For *F. candida*, a minute decrease in terms of the reproduction was observed in animals exposed for 28 days to Fe_3_O_4_ PEG-PLGA [r^2^ = 0.06, hence, not relevant and merelly informative; reproduction EC10 = 492 (−77–1061) mg Fe/kg soil, model: logistic 2 parameters, slope = 4.59 × 10^−3^, top point = 772] (Figure 2).

**Figure 3:**
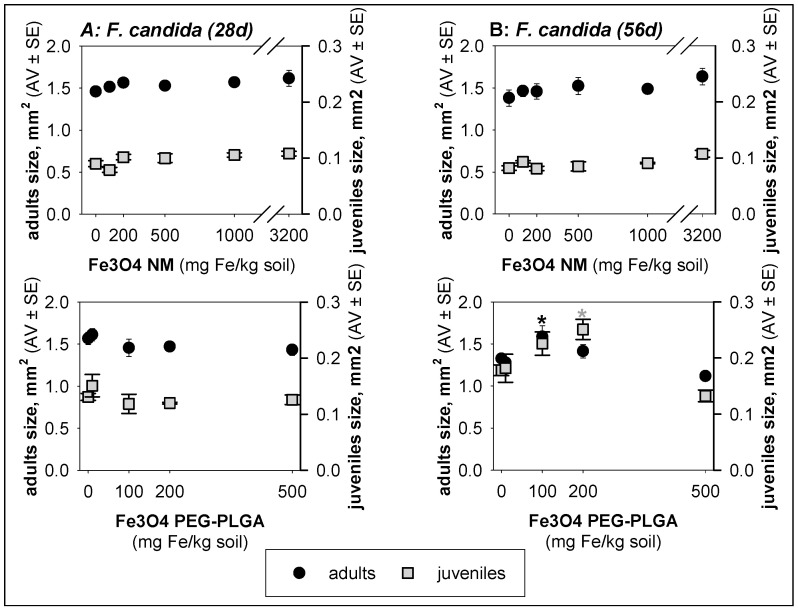
Results in terms of size for *Folsomia candida* exposed to Fe_3_O_4_ NM and Fe_3_O_4_ PEG-PLGA in LUFA 2.2 soil for (**A**) 28 days and (**B**) 56 days. The values are expressed as the average ± standard error (AV ± SE). * *p* < 0.05 (Dunnett’s).

Exposure to Fe_3_O_4_ PEG-PLGA for two generations (56 days) caused a significant (*p* < 0.05) increase in the size of *F. candida* adults (100 mg Fe/kg soil) and juveniles (100 and 200 mg Fe/kg soil) (Figure 3).

Although exposure to Fe_3_O_4_ NMs did not seem to impact survival and reproduction during the 28 days, the results on day 56 show a relative decrease (ca. 17%), whereas, after exposure to Fe_3_O_4_ PEG-PLGA, a relative increase (25–35%) was observed on day 56 (Figure 2C).

## 4. Discussion

Neither of the Fe materials tested were significantly toxic to *E. crypticus* or *F. candida* up to concentrations of 500 mg Fe/kg for Fe_3_O_4_ PEG-PLGA or 3200 mg Fe/kg for Fe_3_O_4_ NM. This observed lack of toxicity is an important result for the risk assessment of these materials and their introduction to the market. It is well-known that non-significant results can disprove an existing hypothesis that these iron-based NBMs cause a substantial negative effect on nature. Testing two species from two different (and highly ubiquitous) organisms showed no effect in the standard or in the extended-duration test. Currently, the literature data on the ecotoxicity of Fe NMs to soil ecosystems are very limited. The earthworm *Eisenia fetida* has been shown to avoid a natural soil spiked with 1.5% Fe_3_O_4_ NM (20–50 nm, Shanghai Aladdin Biochemical Technology Co., Ltd., Shanghai, China) [28]. Further, in the same study, it was shown that Fe_3_O_4_ NMs caused oxidative stress (changes in catalase (CAT), peroxidase (POD), and superoxide dismutase (SOD) activities) and damage (lipid peroxidation) to the worms, but this result was achieved via a filter paper contact test [28] and not through soil exposure.

Although speculative, our results indicate that *F. candida* might be slightly more sensitive to Fe_3_O_4_ PEG-PLGA, as a tendency to reduce reproduction rates was observed after 28 days of exposure, at 500 mg Fe/kg, although no effect was observed after 56 days. It is possible that oxidative stress contributed to those effects, as it was shown that Fe_3_O_4_ PEG-PLGA was able to produce OH radicals in water. Oxidative stress generation by (un-coated, synthesized) Fe_3_O_4_ NM was also reported to occur in the snail *Cornu aspersum* exposed to this NM through food and to the fish *Danio rerio* and *Carassius gibelio* exposed through water [29]. However, in *F. candida*, if occurring, oxidative stress was probably transient, or the organisms were able to cope with this under the given conditions (likely optimal growth conditions), as no effects were observed in the exposure of the second generation (56 days). In fact, Fe_3_O_4_ NMs and Fe_3_O_4_ PEG-PLGA seem to promote reproduction in *E. crypticus*, as a small increase in reproduction performance was observed at lower concentrations, and, in *F. candida*, when exposed to the material for the second generation, an increase in size was observed for the intermediate concentrations. This is similar to an hormesis effect, possibly because Fe is an essential element in living organisms (a micronutrient required for metabolic processes across living organisms such as energy production, DNA repair and replication, regulation of gene expression, etc.). Hormesis effects in response to nanoforms of essential metals are relatively well described in plants, as reviewed by Kolbert et al. [30]. The range of concentrations that promote for instance, growth, are, in fact, those with potential usage in agricultural applications [30].

In summary, the current results seem to not indicate unacceptable environmental risks of the Fe_3_O_4_ PEG-PLGA NBM and its precursor, the Fe_3_O_4_ NM, which is a discovery aligned with the low occupational risk analysis reported in [14]. However, further long(er)-term effects, e.g., multigenerational or full life span, cannot be excluded. Further, the oxidative effect of these particles noticeably indicates some kind of interaction with the surrounding environment; however, a soil medium may be so complex that such effects are blurred.

## 5. Conclusions

The NBM Fe_3_O_4_ PEG-PLGA and its precursor Fe_3_O_4_ NM present no unacceptable risk to the soil invertebrate model species *Enchytraeus crypticus* and *Folsomia candida* based on the standard OECD test (28 days) and its extended-exposure version (56 days).

## Figures and Tables

**Table 1:** Summary of the characterization results from the dynamic light scattering (DLS) analysis of hydrodynamic diameter (Zeta-average) and surface charge (Zeta-potential) for Fe_3_O_4_ NM and Fe_3_O_4_ PEG-PLGA aqueous suspensions, and dissolution results for Fe_3_O_4_ PEG-PLGA. PDI: polydispersity index.

Sample	Conc. (mg/L)	Hydrodynamic Diameter Z-Average (nm)	PDI	Surface Charge Z-Potential (mV)	%Release Fe^2+/3+^/Fe_3_O_4_
Fe_3_O_4_ NM	50	6273 ± 3243	0.9	−28 ± 3	-
	256	3459 ± 1146	1.0	−17 ± 0.7	-
Fe_3_O_4_ PEG-PLGA	50	74 ± 0.4	0.1	−50 ± 3	^1^
	256	76 ± 2	0.2	−49 ± 2	^1^

^1^ below the detection limit of 0.01 mg/L.

## Data Availability

Data will be made available upon request.

## Supplementary Materials

The following supporting information can be downloaded at https://www.mdpi.com/article/10.3390/jox14010017/s1: Table S1: Summary of characterization results from the dynamic light scattering (DLS) test on hydrodynamic diameter (Zeta-average) and surface charge (Zeta-potential) for Fe_3_O_4_ NM aqueous suspensions. PDI: polydispersity index; Table S2: OH radicals formation by means of NBM Fe_3_O_4_ PEG-PLGA. LOD: limit of detection; Table S3: Soil pH (1:5 soil: 0.01 M CaCl_2_) as measured over the toxicity tests performed with *Enchytraeus crypticus*; Table S4: Soil pH (1:5 soil: 0.01 M CaCl_2_) as measured over the toxicity tests performed with *Folsomia candida*; Figure S1: Results in terms of size for *Enchytraeus crypticus* exposed to Fe_3_O_4_ NM for 28 days, in LUFA 2.2 soil. The values are expressed as the average ± standard error (AV ± SE). Figure S2: Results in terms of the number of animals (adults + juveniles) when exposing *Enchytraeus crypticus* in LUFA 2.2 soil to Fe_3_O_4_ NM and Fe_3_O_4_ PEG-PLGA over time, on days 7, 14, 21, 28, and 56. The values represent the population as the average ± standard error (AV ± SE). Figure S3: Results in terms of the number of animals (adults + juveniles) when exposing *Folsomia candida* in LUFA 2.2 soil to Fe_3_O_4_ NM and Fe_3_O_4_ PEG-PLGA over time, on days 7, 14, 21, 28, and 56. The values represent the population as the average ± standard error (AV ± SE).
