# Supplementary material for: Iron Oxide (Magnetite)-Based Nanobiomaterial with Medical Applications—Environmental Hazard Assessment Using Terrestrial Model Species
Source: J Xenobiot. 2024 Feb 22;14(1):285–94. doi: 10.3390/jox14010017 (PMC10971733; doi:10.3390/jox14010017)
Supplement: Supplementary file 1 [file jox-14-00017-s001.zip › jox-2865006-supplementary.pdf]

Supplementary Materials

Table S1: Summary of characterization results from the Dynamic Light Scattering (DLS) on hydrodynamic diameter (Zeta average) and surface charge (Zeta potential) for Fe3O4 NM aqueous suspensions. PDI: polydispersity index.

**conc.** (mg/L)	**Hydrodynamic diameter** Z-average (nm)	**PDI**	**Surface charge** Z-potential (mV)
3200	4550 ± 1003	0.4	-13.9 ± 0.76
1000	6144 ± 915.5	0.9	-12.1 ± 0.51
500	5203 ± 423.6	0.8	-13.7 ± 1.62
200	5582 ± 1637	0.7	-22.1 ± 0.45
100	3985 ± 941.9	1.0	-29.2 ± 2.57

Table S2: OH Radicals formation by means of NBM Fe_3O_4 PEG-PLGA. LOD: limit of detection.

**Sample**	**2.5 DBHA (nM)**	**Time (h)**	**2.5 DBHA production rate (nM h^{-1})**	**$OH^{\cdot}$ production rate (nM h^{-1})**
Blank	<LOD	24	<LOD	<LOD
Fe_3O_4 PEG-PLGA	139	24	5.8	21

Table S3: Soil pH (1:5 soil: 0.01M $CaCl_2$) as measured over the toxicity tests performed with *Enchytraeus crypticus.*

NM	Conc. (mg Fe/kg soil)	Time (days)		
		0	28	56
Fe_3O_4	0	5.3	5.6	5.2
	100	5.4	5.6	5.0
	200	5.4	5.5	5.0
	500	5.4	5.5	4.9
	1000	5.4	5.5	4.9
	3200	5.4	5.5	5
Fe_3O_4 PEG-PLGA	0	5.3	5.4	4.5
	0-disp	5.2	5.0	4.5
	10	5.2	5.0	4.5
	100	5.2	5.1	4.5
	200	5.4	5.1	5.0
	500	5.4	5.0	5.1

Table S4: Soil pH (1:5 soil: 0.01M $CaCl_2$) as measured over the toxicity tests performed with *Folsomia candida.*

NM	Conc. (mg Fe/kg soil)	Time (days)		
		0	28	56
Fe_3O_4	0	5.1	5.5	5.6
	100	4.9	5.5	5.3
	200	4.8	5.4	4.8
	500	4.8	5.4	4.7
	1000	4.9	5.2	5.2
	3200	4.9	5.4	4.9
Fe_3O_4 PEG-PLGA	0	5.1	5.2	4.4
	0-disp	5.1	5.1	4.4
	10	5.1	5.1	4.4
	100	5.1	5.0	4.4
	200	5.0	5.1	4.4
	500	4.7	5.2	4.5

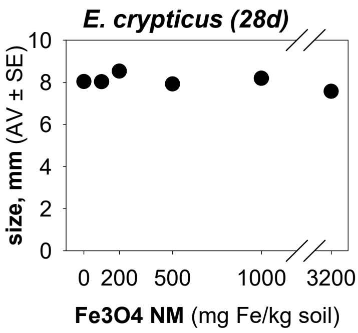

Figure S1: Results in terms of size for *Enchytraeus crypticus* exposed to Fe_3O_4 NM for 28 days, in LUFA 2.2 soil. Values are expressed as average ± standard error (AV ± SE).

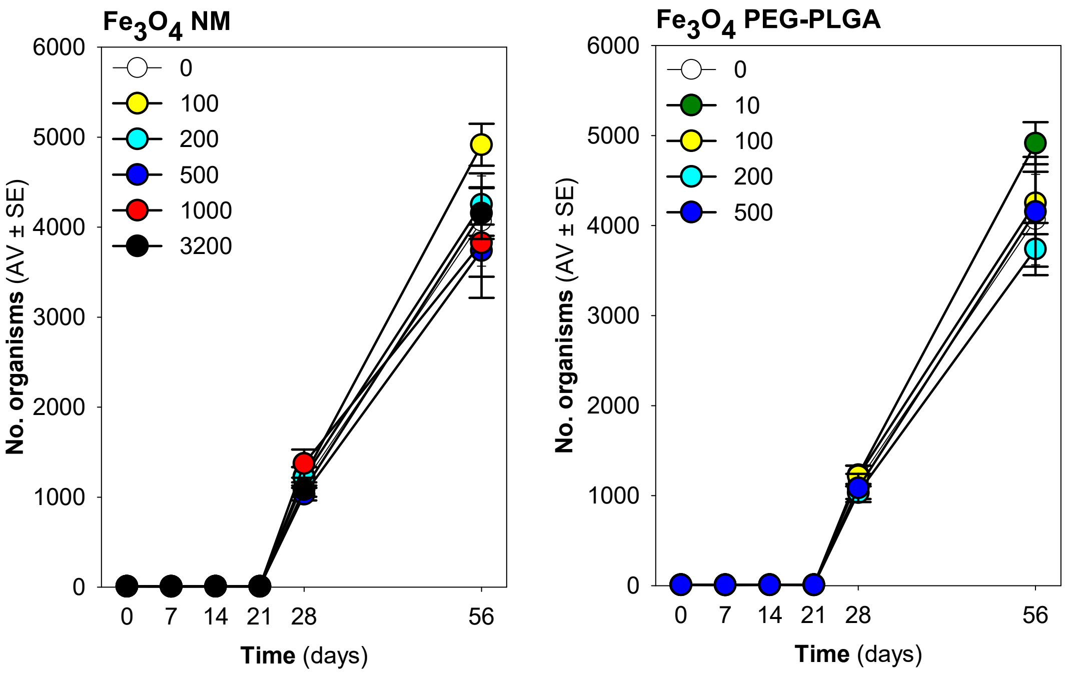

Figure S2: Results in terms of number of animals (adults + juveniles) when exposing *Enchytraeus crypticus* in LUFA 2.2 soil to Fe_3O_4 NM and Fe_3O_4 PEG-PLGA over time, at days: 7, 14, 21, 28 and 56. Values represent population as average ± standard error (AV ± SE).

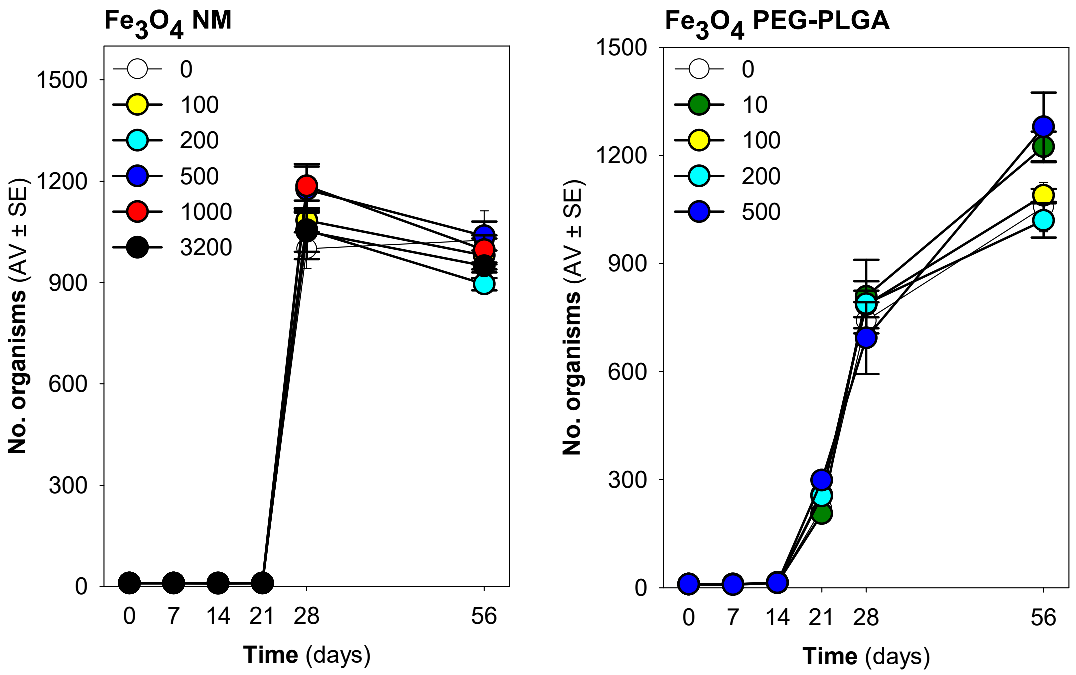

Figure S3: Results in terms of number of animals (adults + juveniles) when exposing *Folsomia candida* in LUFA 2.2 soil to Fe_3O_4 NM and Fe_3O_4 PEG-PLGA over time, at days: 7, 14, 21, 28 and 56. Values represent population as average ± standard error (AV ± SE).
